# Differences in swimming ability and its response to starvation among male and female *Gambusia affinis*

**DOI:** 10.1242/bio.022822

**Published:** 2017-04-10

**Authors:** Jiangtao Li, Xiaotao Lin, Zhongneng Xu, Jun Sun

**Affiliations:** Institute of hydrobiology, Jinan University; Engineering Research Center of Tropical and Subtropical Aquatic Ecological Engineering, Ministry of Education, Guangzhou, Guangdong 510632, China

**Keywords:** Invasive fish, Sexual dimorphism, Energy metabolism, Burst swimming speed, Critical swimming speed

## Abstract

To explore the differences in the swimming ability and environmental adaptive abilities between male and female *Gambusia affinis*, we assessed the differences in burst swimming speeds (*U*_burst_), critical swimming speeds (*U*_crit_) and their related fin areas, and consumption of energy substances after starvation at 0 (control group), 15, 30, 45, and 60 days, respectively. The results showed that the pectoral and caudal fin areas did not differ significantly between male and female *G. affinis*. However, the dry mass, condition factors, and absolute contents of glycogen, lipids, and proteins were significantly elevated in females in the control group (*P*<0.05), whereas *U*_burst_ and *U*_crit_ were significantly low (*P*<0.05). After starvation of 60 days, the rate of consumption of lipids was significantly low in the females (*P*<0.05). Although *U*_burst_ and *U*_crit_ decreased linearly with increased duration of starvation, the coefficient of linear equation between *U*_crit_ and starvation time was significantly lower in females than males (*P*<0.05). These findings indicated that low body mass and condition factors reduce the relative bear load and moving resistance that causes high swimming performance in male *G. affinis*. High contents of energy substances and low rate of consumption of lipids result in stable *U*_crit_ in females during hunger.

## Abstract (precis)

**Summary:** The study explored the causes of variation in swimming performance with respect to morphological and physiological characteristics, which provides further information about environmental adaptive ability in *Gambusia affinis*.

## INTRODUCTION

Swimming performance is crucial for the survival of fish in aquatic environments and is considered a crucial determinant of the fitness of fish (Oufiero and Garland, 2009). The speed of swimming of fish is primarily categorized into burst swimming speed (*U*_burst_) and critical swimming speed (*U*_crit_) according to the levels of oxygen demand. Burst swimming is powered anaerobically by white muscle and is considered as the highest speed; however, it is used only for a short period (<15 or 20 s), especially while escaping from predators, catching the prey, and passing rapids, riffles, and fishways (Kieffer, 2000; Osachoff et al., 2014; Yeh et al., 2010). Critical swimming is driven by aerobic conditions using the red muscle for a prolonged period without fatigue (Brett, 1972). *U*_crit_ is the optimum speed of swimming of the fish during routine activities, such as cruising and finding mates (Langerhans, 2009; Sinclair et al., 2011). The western mosquitofish, *Gambusia affinis*, is a small ovoviviparous fish species of the family Poeciliidae that is native to North America (Caiola and Sostoa, 2005). Previously, several countries had adopted *G*. *affinis* as a biological tool to eliminate mosquitoes; however, due to strong ecological adaptation and reproductive capacity, currently, it is an eminent invasive species worldwide. In China, *G*. *affinis* was found in static water areas such as lakes, ponds, ditches, or in some river areas with a low velocity of the water (Yan et al., 2009). Moreover, in recent years, *G*. *affinis* has been observed invading the mountain streams located in southern China, causing survival pressure to the endangered aboriginal fish (Chen, 2010).

In the mountain streams, the fluidity of water principally embodies high spatial heterogeneity and significant seasonal variation (Yan et al., 2010). The fluctuations in the water velocity are apparent in the mountain streams in southern China as these streams are often washed by heavy rains and flash floods due to subtropical monsoons. Therefore, the swimming ability is crucial for the survival of fish in these areas. The pectoral and caudal fins primarily propel the fish while swimming and are commonly powered by abenosine triphosphate (ATP), which is synthesized by decomposing the energy substances such as glycogen, lipids, and proteins (Marras et al., 2010; Reidy et al., 2000; Tu et al., 2011). Therefore, the swimming ability in some fishes may differ in both sexes due to differences in fin size and/or energy metabolism; these differences are caused by various selection pressures during long-term evolution. Furthermore, in mountain streams, the volume of animal baits such as zooplankton is less and seasonal variations are more than that observed in the static water conditions (Yuan and Luo, 2003). Thus, the fish inhabiting such mountainous areas often lack food. Previous studies suggested that the swimming performance of the fish is reduced to a certain degree after starvation that might be attributed to the decreasing levels of enzyme activity, which, in turn, is related to swimming (Faria et al., 2010; Killen et al., 2014; Martinez et al., 2004). Fish can only rely on the decomposition of glycogen, lipids and proteins stored in the body to gain energy for swimming and perform other essential life activities when food is insufficient (Lin, 2011; Machado et al., 1988). Therefore, the decrease in the swimming ability after hunger might correlate to the storage of energy substances and their metabolic characteristics. Further exploration regarding this association could provide an insight into the hunger-resistant ability of *G*. *affinis* and adaptability to the mountain streams. However, previous studies on the swimming performance of *G*. *affinis* were primarily focused on the effects of fin damage, age, pregnancy and predation pressure on swimming ability and the differences between wild and non-wild fish (Belk and Tuckfield, 2010; Langerhans et al., 2005, 2004; Plaut, 2002; Sinclair et al., 2011; Ward et al., 2003). In addition, most of these studies utilized either males or females as experimental models. Whether there are differences in the swimming performances between the male and female *G*. *affinis* and the response to starvation are yet unclear. Therefore, in the present study, we used male and female *G*. *affinis* at first sexual maturity to assess the gender-associated differences in burst swimming speeds (*U*_burst_), critical swimming speeds (*U*_crit_), the related morphological characteristics, and consumption of energy substances after different starvation conditions. This study aimed to examine the causes of differences in swimming performance between male and female *G*. *affinis* with respect to morphological and physiological characteristics. The results provided additional information on the adaptive ability of *G. affinis* to mountain streams and the influence of sexual differences in daily activities.

## RESULTS

### Morphological characteristics

The values of morphological characteristics were summarized in Table 1. No significant interactions between starvation times and genders were observed with respect to body lengths; however, extremely significant interactions (*P*<0.01) were found in dry masses and condition factors. Further comparisons revealed that although the body lengths of both male and female *G*. *affinis* did not vary significantly during different starvation times; significant variations (*P*<0.05) were observed in dry mass and condition factors after starvation and all the indicators decreased with starvation time. No significant differences were recorded in body lengths between male and female *G. affinis* at any point in the time of starvation. The dry mass and condition factors were significantly higher in females than males (*P*<0.05) at 0-15 days of starvation; however, these values showed no significant differences between males and females at 30-60 days of starvation.

**Table 1.**
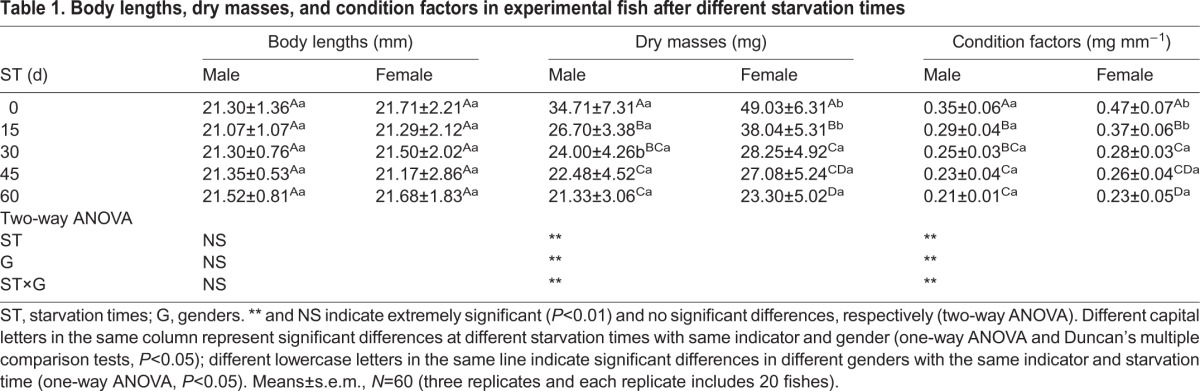
**Body lengths, dry masses, and condition factors in experimental fish after different starvation times**

**Fig. 1.**
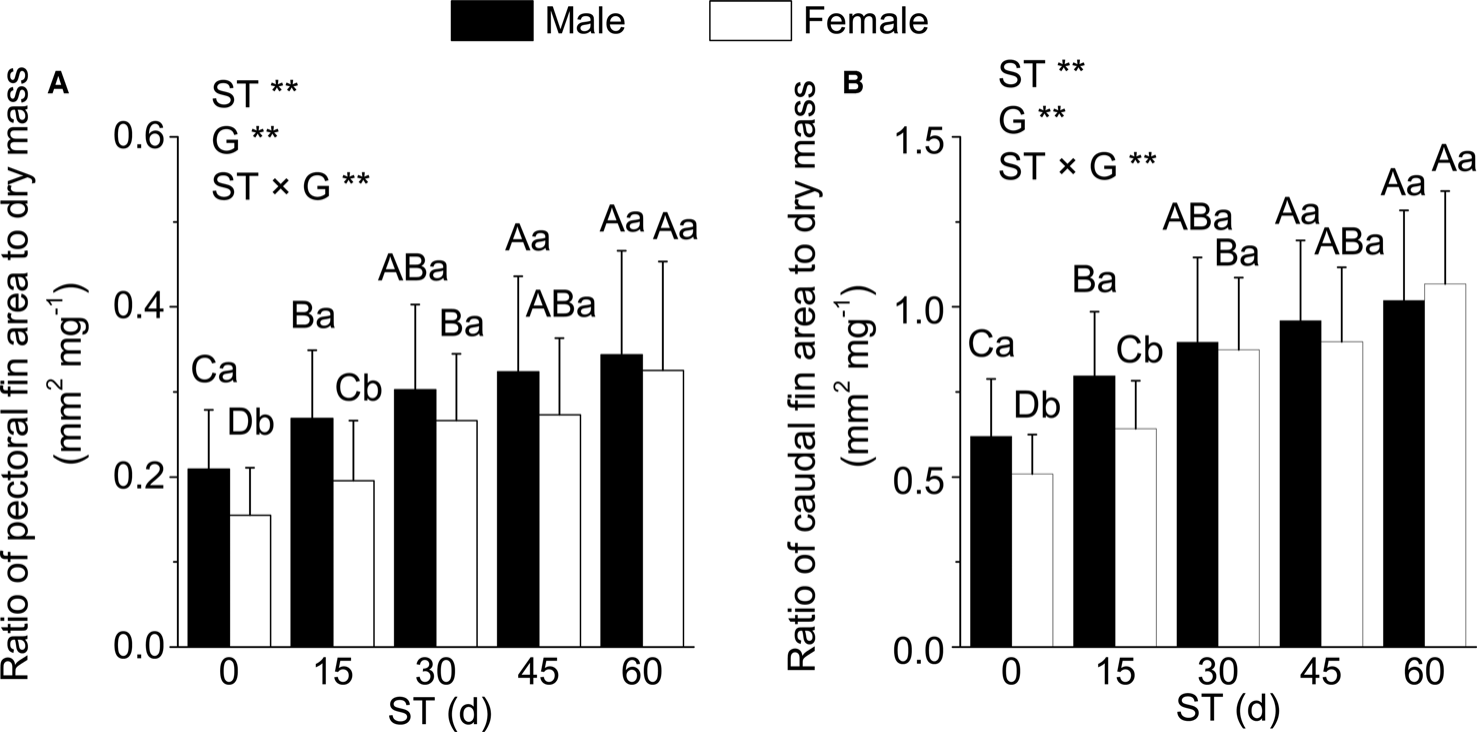
**Comparison of the ratio of fin area to dry masses in male and female fish.** (A) Pectoral fin. (B) Caudal fin. ST, starvation times; G, genders. ** indicates extremely significant differences (two-way ANOVA, *P*<0.01). Different capital letters represent significant differences at different starvation times in the same gender (one-way ANOVA and Duncan's multiple comparison tests, *P*<0.05); different lowercase letters represent significant differences in the different genders at same starvation time (one-way ANOVA, *P*<0.05). Means±s.e.m., *N*=60 (three replicates and each replicate includes 20 fishes).

The variation scope of pectoral fins areas was 7.40±2.44-7.59±2.74 mm^2^ (female) and 7.18±2.14-7.33±2.61 mm^2^ (male) in all starvation conditions. The variation scope of caudal fins areas was 24.30±5.93-24.92±5.7 mm^2^ (female) and 21.28±5.04-21.73±5.66 mm^2^ (male) in all starvation conditions. Furthermore, neither significant differences between different starvation times nor between different genders, and no significant interactions between starvation times and genders were observed while evaluating using the two-way ANOVA in pectoral fins areas. Similar results were also noted for the caudal fin areas. However, when the pectoral and caudal fin areas of *G*. *affinis* were considered together with dry mass, the ratio of the pectoral fin area to dry mass and the ratio of the caudal fin area to dry mass were both significantly higher in males than females at 0-15 days of starvation (*P*<0.05), but no significant differences were observed with respect to gender at 30-60 days of starvation (Fig. 1).

### Consumption of energy substances

The content of energy substances can be represented by relative contents (content per unit of body mass, %) or absolute contents (content per individual fish, mg ind^−1^). The relative contents were calculated by the percentage of each energy substance, and the values of energy substances (especially proteins) might increase after starvation due to the decrease in body mass. The consumption of energy substances can be calculated accurately by decreasing the absolute content rather than the relative content, as the relative content reflects the variation of relative ratio rather than the amount of consumption of each energy substance. Therefore, the absolute contents were used widely to examine the effects of starvation on fishes in previous studies (Simpkins et al., 2003; Urzúa et al., 2013). Thus, in the present study, we used the absolute content (mg ind^−1^), absolute consumption amount (mg), and absolute consumption rate (%) to assess the consumption of energy substances, although the body mass in females was higher than that in males during no starvation.

The absolute contents of energy substances were listed in Table 2. Extremely significant (*P*<0.01) and significant (*P*<0.05) interactions between starvation times and genders were observed with respect to glycogen and lipid contents, respectively. However, no significant interactions between the starvation times and genders were observed for protein content. Further comparisons showed that the contents of glycogen, lipids and proteins altered significantly after starvation in both male and female *G. affinis* (*P*<0.05). The glycogen content was significantly higher in females than the males at 0 days of starvation (*P*<0.05); however, no significant differences were observed between males and females at 15-60 days of starvation, which was in proximity zero. The contents of lipids and proteins were significantly higher in females as compared to males at any point of starvation time (*P*<0.05).

**Table 2.**
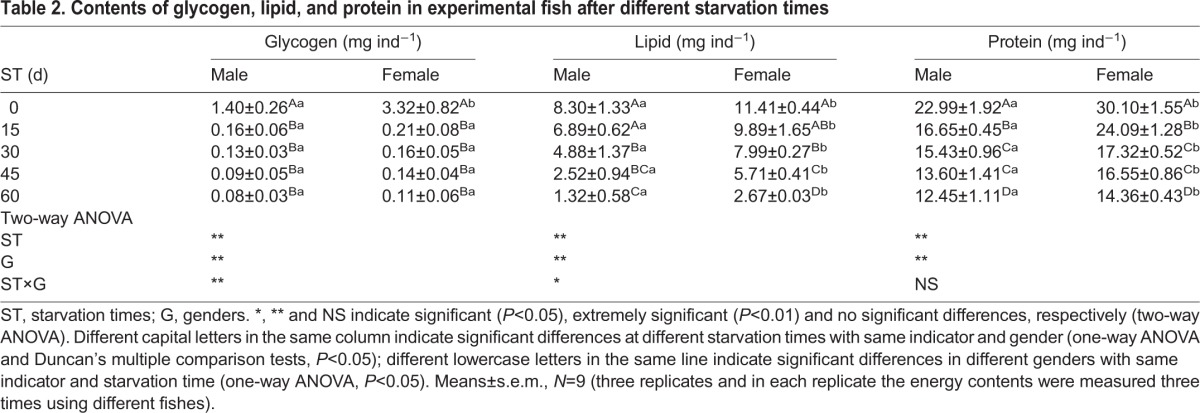
**Contents of glycogen, lipid, and protein in experimental fish after different starvation times**

**Fig. 2.**
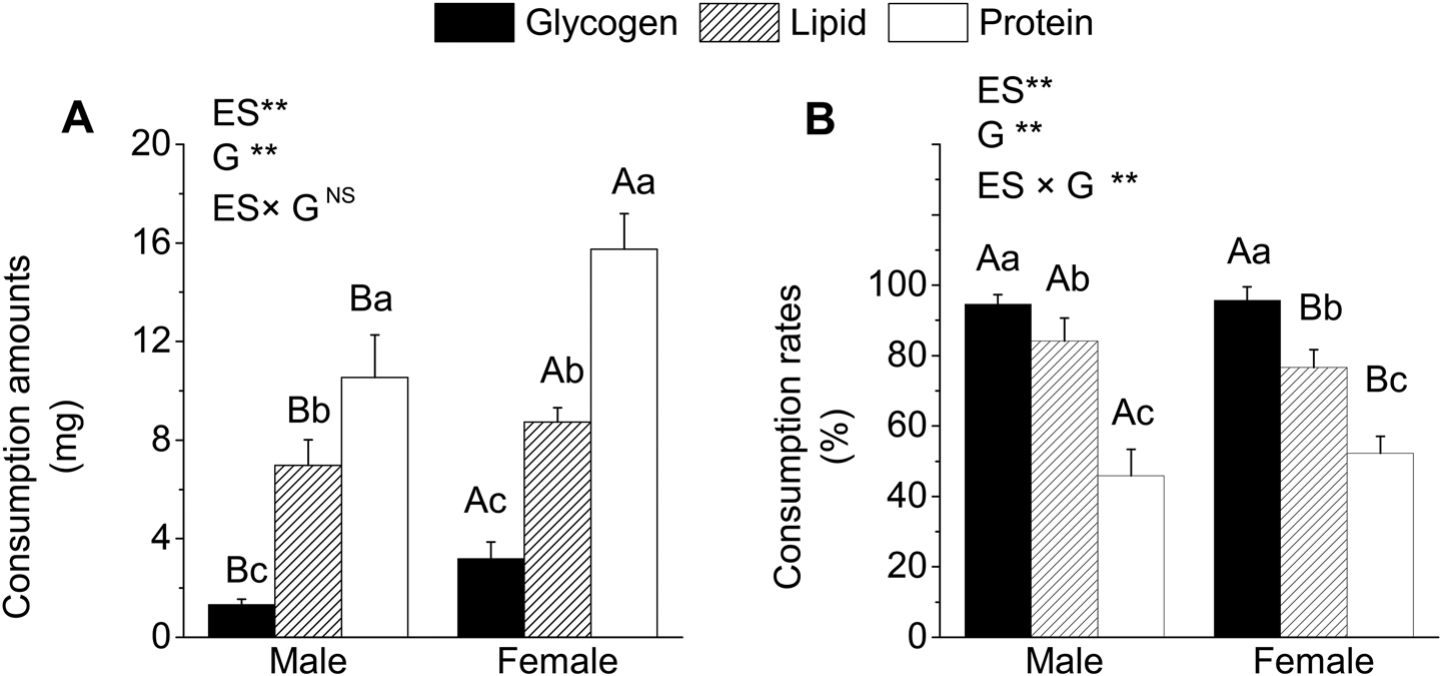
**Consumption of energy substances of male and female fish after 60 days starvation.** (A) Consumption amounts. (B) Consumption rates. ES, energy substances; G, genders. ** and NS represent extremely significance (*P*<0.01) and no significant differences, respectively (two-way ANOVA). Different capital letters indicate significant differences at different genders in same energy substances content (one-way ANOVA, *P*<0.05); different lowercase letters represent significant differences in different energy substances at the same gender (one-way ANOVA and Duncan's multiple comparison tests, *P*<0.05). Means±s.e.m., *N*=9 (three replicates and in each replicate the energy contents were measured three times using different fishes).

The amounts and rates of consumption of energy substances after the longest starvation for 60 days were shown in Fig. 2. No significant interactions between energy substances and genders were observed with respect to the amount of consumption (Fig. 2A); however, extremely significant interactions (*P*<0.01) were found in the rates of consumption (Fig. 2B). Further analysis showed that the amount of consumption of proteins, lipids and glycogen were the highest, second, and minimum, respectively, in both male and female *G*. *affinis* (Fig. 2A). The amount consumption of lipids, glycogen and proteins was significantly higher in females than males (*P*<0.05; Fig. 2A). However, the trend of the rate of consumption was converse in both male and female, of which glycogen, lipids and proteins were the highest, second, and minimum, respectively (Fig. 2B). No significant differences were observed between male and female in the rate of consumption of glycogen; both showed approximately 100% (Fig. 2B). The rate of consumption of lipids was significantly higher in males as compared to females (*P*<0.05; Fig. 2B); however, that of proteins was opposite and significantly higher in females than males (*P*<0.05; Fig. 2B).

### Variation in swimming performance

Significant (*P*<0.05) and extremely significant (*P*<0.01) interactions between starvation times and genders were observed in *U*_burst_ and *U*_crit_, respectively (Table 3). Further comparisons showed that *U*_burst_ and *U*_crit_ varied significantly (*P*<0.05) with increased starvation time in both males and females, and these values decreased after starvation (Table 3).

**Table 3.**
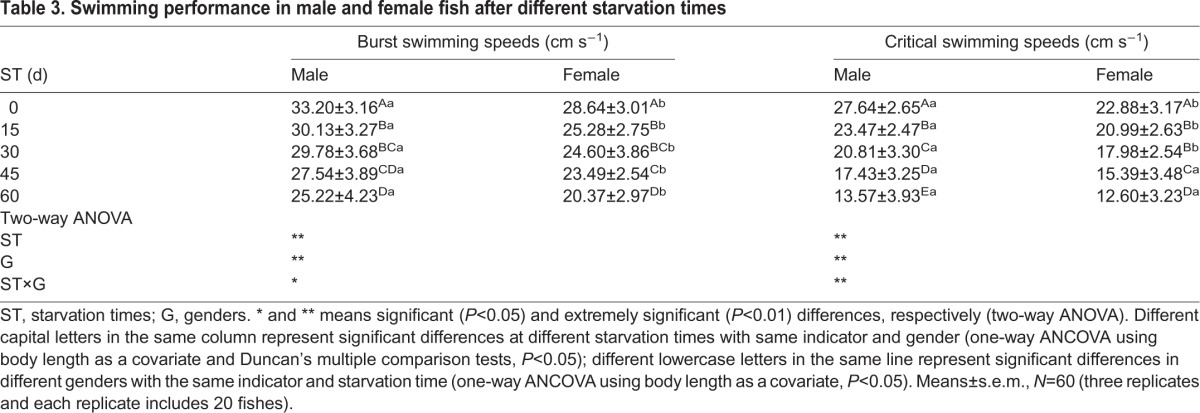
**Swimming performance in male and female fish after different starvation times**

**Fig. 3.**
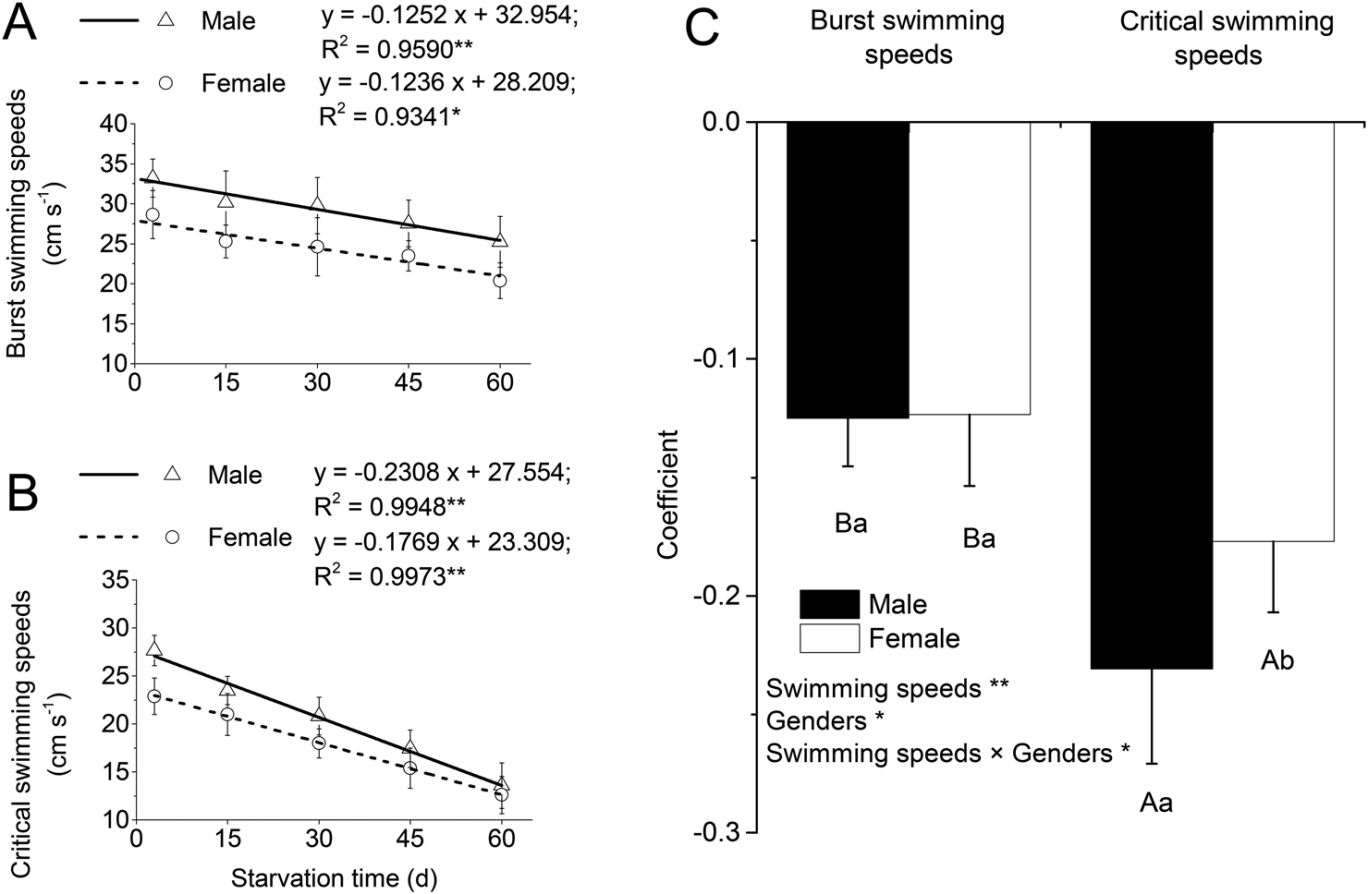
**Relationship between swimming performance and starvation time in male and female fish.** (A) Burst swimming speeds. (B) Critical swimming speeds. (C) The comparison with the coefficient. * and ** indicate significant (*P*<0.05) and extremely significant (*P*<0.01) relevance (A and B: ANCOVA) or differences (C: two-way ANOVA), respectively. Different capital letters in C indicate significant differences at different swimming speeds in the same gender (one-way ANOVA, *P*<0.05); different lowercase letters in C indicate significant differences in different genders at same swimming speed (one-way ANOVA, *P*<0.05). Means±s.e.m., *N*=3 (three replicates).

*U*_burst_ and *U*_crit_ decreased linearly with starvation time (Fig. 3A,B). The comparison of coefficients was shown in Fig. 3C. Significant interaction were observed between swimming speeds and genders in the coefficient of linear equation (*P*<0.05; Fig. 3C). Further comparisons showed that the coefficient of linear equation between *U*_burst_ and starvation time was significantly lower than that of the equation between *U*_crit_ and starvation time in both male and female (*P*<0.05; Fig. 3C). Furthermore, no significant differences were observed between male and female in the coefficient of linear equation between *U*_burst_ and starvation time; however, in the case of *U*_crit_, it was significantly lower in females than males (*P*<0.05; Fig. 3C).

## DISCUSSION

### Swimming performance in fish during non-starvation

The morphological differences between male and female species occurred in some fishes because different genders encountered different selection pressures in long-term evolution, such as fecundity, sexual, and niche differentiation selections (Chu and Lee, 2012; Elgar, 1990). The differentiation of morphological characteristics that were associated with swimming can cause the differences in swimming ability between male and female fishes (Li et al., 2016). The fins of the fishes are the most vital organs for swimming, and thus, the size substantially affects the swimming performance as fins transfer a large proportion of propulsive power created by the muscles into the water (Plaut, 2000). Caudal and pectoral fins can provide the forward force, and also can control the direction, lift, and the balance of the fish body during swimming (Liu et al., 2014; Plaut, 2000). Our previous study showed that the size of caudal and pectoral fins are related to the swimming ability as the large size of caudal and pectoral fins can accelerate vast amounts of water and facilitate propulsion during swimming (Li et al., 2016). In this study, both areas of pectoral fins and caudal fins were not significantly affected by genders, suggesting that areas of caudal or pectoral fins did not differ significantly between male and female *G*. *affinis*; however, *U*_burst_ and *U*_crit_ were significantly higher in males than females (Table 3). These results could not be explicated solely by propulsion that was produced by the caudal and pectoral fins, rather the propulsion per unit of body mass, the difference in swimming performance caused by different body masses, and physical features between male and female *G*. *affinis*. Previous studies have shown that the increased body mass and maximum cross-section during the pregnancy of female *G*. *affinis* was one of the causes that led to the decrease in the swimming performance, although parts of oxygen were absorbed in the gill and used by the embryos in pregnant female (Plaut, 2002). In the present study, the male and female *G*. *affinis* were fed separately after the gender was distinguished in order to avoid female pregnancy that would affect the subsequence test of swimming performance. Thus, the excessive oxygen consumption due to the embryos was not a reason for the reduction in *U*_burst_ and *U*_crit_ in female *G*. *affinis*. Therefore, in our study, during no starvation, *U*_burst_ and *U*_crit_ were significantly lower in non-pregnant females than those in males, which might be due to higher body mass and condition factors in females. The higher body mass and condition factors, which indicate obesity and high maximum cross-section, definitely result in an increased resistance during swimming. On the other hand, this phenomenon also indicates that relatively less propulsion per unit of body mass was produced by the same size fin, and that decreased the swimming speeds. Therefore, in our study, when the fin size of *G*. *affinis* was considered together with dry mass, both the ratios of pectoral fin areas to dry mass and ratios of caudal fin areas to dry mass were significantly higher in males than females (Fig. 1), which might be attributed to the characteristic elevated swimming performance in males.

The locomotor speeds in most males are faster than that in females as males are predisposed to expose themselves often during mating or territorial pursuit (Van Damme et al., 2008). *U*_crit_ is commonly known to participate in the aerobic exercises such as cruising and finding mates (Langerhans, 2009; Sinclair et al., 2011), whereas *U*_burst_ is involved during fast movement such as escaping from predator and catching preys, which is crucial for survival (Osachoff et al., 2014). Male *G*. *affinis* are required to find mates for active copulation (Pyke, 2005), and the number of males is lower than that of females at sexual maturity in wild habitats (Zheng and Pan, 1985). Therefore, higher *U*_burst_ and *U*_crit_ is advantageous to male *G*. *affinis* for finding mates, predatory activities, escaping from predators, and also in maintaining a high rate of survival to balance the number and stability of the population of the species.

### Consumption of energy substances during starvation

The current experiment showed that at 0 and 15 days of starvation, the ratio of pectoral fin areas to dry mass and the ratio of caudal fin areas to dry mass were both significantly higher in males than females. However, at 30, 45 and 60 days of starvation, the values showed no significant differences between males and females. Therefore, the propulsion per unit of body mass produced by the swinging of caudal and pectoral fins might not cause the differences in the swimming performances between males and females after a particular starvation period. The variations in the swimming performances after starvation were caused by the physiological responses to hunger, and our study aimed to explore the mechanism underlying the storage and consumption of energy substances.

The loss of body mass and condition factors was approximately 50% in *Hoplias malabaricus* after 180 days of starvation (Rios et al., 2002), suggesting that life activities were powered by energetic substances stored in the body of the fish during hunger. Although there is a great variability in the source and quantity of energy available, the energy for life activities must be derived from the catabolism of lipids, carbohydrates and proteins (Weber, 2011). However, the consumption and utilization of lipids, carbohydrates and proteins during starvation were different in different fishes. Primarily, the glycogen and lipids were consumed, but the protein intake was less in most of the fishes (Kutty, 1978). Furthermore, the consumption of lipids was a priority, followed by the utilization of glycogen and proteins in *Dicentrarchus labrax* and *Pleuronectes platessa* (Jobling, 1980; Stirling, 1976). In the present study, the absolute content of glycogen was decreased rapidly during starvation and was approximately zero at 15 days of starvation; however, the absolute contents of lipids and proteins were decreased gradually with increased starvation time (Table 2). After 60 days of starvation, the amount of protein consumption was at the highest, followed by lipids and glycogen in both males and females (Fig. 2A). Contrastingly, the rate of consumption of glycogen was highest and reached approximately 100%, followed by lipids, which was about 76.63-84.15%, and proteins that was about 45.84-52.31% (Fig. 2B). These results indicated that both male and female *G*. *affinis* were mainly powered by consumption of proteins and lipids for life activities during the later period of starvation because glycogen had been almost exhausted in the early period of starvation. The higher rate of consumption of lipids than proteins suggested that *G*. *affinis* preferentially consumes the lipids to fuel the fish body and proteins during starvation*.* The activity metabolism can be up to 10–15-fold more than the basic metabolism during exercises of different intensities (Brett, 1972), indicating that the fish needs to expend more energy during the swimming process. Thus, the consumption and storage of energy substances can cause variations in the swimming performance during the starvation period.

### Relationship between energy supply and swimming performance after starvation

The exercise is known to be powered primarily by ATP in the muscles at the early stage of movement. The amount of ATP stored in the muscles is low, and during subsequent stages of the movement, the fish fulfills the increased demands of ATP required during muscle contraction through hydrolysis of phosphocreatine (PCr), glycolysis and aerobic metabolism (Li et al., 2015; Richards et al., 2002). ATP is synthesized in many ways due to different movements. ATP for anaerobic exercise is mainly synthesized by the anaerobic metabolism, such as the hydrolysis of PCr and glycolysis (Deng et al., 2007; Richards et al., 2002). On the other hand, the ATP for aerobic movements is mainly synthesized by oxygenolysis of glycogen, lipid and protein (Li et al., 2015; Wilmore and Costill, 1994; Zhu et al., 2016), although the hydrolysis of PCr, glycolysis and aerobic metabolism was not independent and synthesized ATP together (Deng et al., 2007). Burst swimming was powered anaerobically by white muscle while critical swimming was principally fueled aerobically, although it may incorporate both aerobic and anaerobic muscles (Bone et al., 1978; Yeh et al., 2010). Therefore, the variations in *U*_burst_ and *U*_crit_ may be different after starvation in most of the fishes. Martinez et al. (2004) found that the *U*_burst_ of *Gadus morhua* was more stable than *U*_crit_ after 12 weeks of starvation, and the declined percentage of *U*_burst_ (30%) was lower than that of *U*_crit_ (38%). In the present study, the *U*_burst_ and *U*_crit_ in both male and female *G*. *affinis* was decreased in a linear manner with respect to starvation time and the coefficient of the linear equation between *U*_burst_ and starvation time was significantly lower than that of the equation between *U*_crit_ and starvation time (Fig. 3). This phenomenon indicated that *U*_burst_ was more stable than *U*_crit_ during starvation, which is also in agreement with the previous results by Martinez et al. (2004); however, the underlying factors might be related to the different ways in which energy was supplied. Burst swimming was powered by white muscle anaerobically, and the energy was first supplied by ATP stored in the muscle, followed by hydrolysis of PCr and glycolysis. Critical swimming principally relies upon red oxidative fibers and is more closely related to the stored amounts of glycogen, lipids and proteins. In the present study, the values of glycogen, lipids and proteins were significantly decreased after starvation (Table 2). A previous study demonstrated that the amounts of PCr in muscles are stable and the storage amounts did not significantly decline after starvation (Kieffer and Tufts, 1998), which could be ascribed to stable *U*_burst_ than *U*_crit_ after starvation. Due to the involvement in escaping from the predators, catching prey, or passing through rapids riffles (Kieffer, 2000; Osachoff et al., 2014), *U*_burst_ may be more important than *U*_crit_ for survival in most fishes. Therefore, we speculate that male and female *G*. *affinis* encompassed stable *U*_burst_ after starvation, which was due to natural selection and evolution, ensuring that *G*. *affinis* continually showed viability under conditions of starvation.

However, our study showed that the coefficient of the equation between *U*_crit_ and starvation time was significantly lower in female than male (Fig. 3C), suggesting that the *U*_crit_ of a female was more stable during starvation. One of the reasons might be due to the storage and consumption of energy substances after starvation. The time-related efficiency of decomposition of glycogen was higher as compared to that of lipids and proteins. The energy for the movement was supplied by decomposing muscle glycogen and hepatic glycogen. In our study, the glycogen in the whole fish body was almost exhausted at the early stage of starvation, and then, the energy for *U*_crit_ was mainly supplied by lipids and proteins at later stages of starvation. Proteins are vital substances, which have lower caloric value than the lipids, although the storage amount is higher in the fish body. Thus, *G*. *affinis* might consume lipids to power *U*_crit_. Therefore, in the present study, higher storage and lower consumption rates of lipids occurred in female *G*. *affinis* than in males, which may be one of the causes for a stable *U*_crit_ in females even after a prolonged period of starvation. This characteristic indicated that the female *G*. *affinis* has stronger anti-hunger ability than males.

In conclusion, the swimming performance and its related morphology and energy metabolism characteristics showed significant differences between male and female *G*. *affinis*. The lower body mass and conditional factors resulted in adequate swimming performances in male *G*. *affinis*, beneficial in mating and stabilizing the population structure. The high contents of energy substances and stable *U*_crit_ indicate a robust anti-hunger ability in females, advantageous in maintaining reproductive output in mountain streams where the food is always scanty. These results can provide further information about the ecophysiological mechanisms underlying the environmental adaptation in habitats, such as mountain streams, among the male and female *G*. *affinis.*

## MATERIALS AND METHODS

### Experimental fish

We declare that the care and use of experimental animals comply with relevant institutional and animal welfare laws of the country where the experiments were performed. All the experimental fish were hatched from adult females. Adult *G*. *affinis* were captured with a net from a mountain stream in Conghua, Guangdong province, China. A large number of adult female fish were maintained individually in an aquarium until reproduction. After birth, the larvae of the fish were raised in the aquarium and fed zooplankton, captured from the pond in Jinan University, from 0–19 days and *Chironomus* sp*.* twice (09:00 h and 21:00 h) daily after 19 days. Standard conditions were maintained, including water temperature at 25±1°C, photoperiod of 14 h light:10 h dark, and illumination intensity 580 lx. One-third of the water in the aquarium was replaced daily and supersaturated with oxygen by continuous aeration. The swimming performance of female *G*. *affinis* has been demonstrated to be reduced during pregnancy (Plaut, 2002). Therefore, when the anal fin began to differentiate and the gender was distinguished (Pyke, 2005), the male and female species were raised separately to avoid pregnancy that would impact the swimming ability. The length of the body of *G*. *affinis* may vary according to the different regions and other experimental conditions; however, the body length of female *G*. *affinis* was approximately 2.1 cm when it reached the first sexual maturity in our laboratory (Zhu et al., 2015). Furthermore, the body lengths of male and female *G*. *affinis* are identical during the initial stages of sexual maturity (Pyke, 2005). Thus, in the present study, when the body lengths of male and female *G*. *affinis* reached 2.1 cm, 1100 fishes each of both sexes were randomly selected as models used for subsequent experiments.

### Experimental protocol

The amounts of phytoplankton and zooplankton are scant in the mountain streams in southern China, especially in January, February and March (Zhou et al., 2016). Therefore, the *G*. *affinis* invading these areas might suffer from hunger stress for a prolonged duration. In addition, a preliminary experiment showed that few *G*. *affinis* appeared dead after 60 days of starvation, and hence, the longest starvation period in the present study was 60 days.

The experimental fish were deprived of feed for 24 h before the start of the experiment. The time of starvation in both male and female was set to 0 (control group), 15, 30, 45 and 60 days, respectively, performed in three replicates. From each replicate, 70 fishes were randomly selected during the first sexual maturity and moved solitarily into a 500-ml glass beaker with 400 ml water for starvation at each point in time. The glass beakers were placed in aquariums, where the temperature was maintained at 25±1°C, and the illumination intensity was 580 lx; two-thirds of the water in the glass beaker was replaced every 3 days. After starvation, 60 fishes from each replicate were divided into two groups, and the remaining fishes were reserved as standby. The first group included 20 fishes, which were used to measure the contents of energy substances in the whole body and the second group including 40 fishes were used for measuring the swimming ability and fin areas.

### Measurement of energy substances

In each replicate, the body lengths of 20 mildly anesthetized (50 mg l^−1^-buffered MS-222) *G. affinis* individuals in the first group were measured individually to the nearest 0.01 mm and dried body masses to the nearest 0.01 mg. These 20 fishes were then used to measure the contents of glycogen, lipids, and proteins in the whole body independently. Each energy substance was measured three times. Each time, two fishes were used for measurement. Lipids were measured using Soxtec Extraction System (Denmark, Foss, 2055), proteins were determined using a Kjeldahl Automatic Analyzer (Denmark, Foss, 2300), and glycogen was evaluated by anthrone-sulfuric acid method.

The related formulas were as follows:

condition factors (%)
(1)
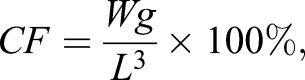
consumption amount of energy substance (mg ind^−1^)
(2)
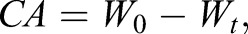
consumption rate of energy substance (%)
(3)
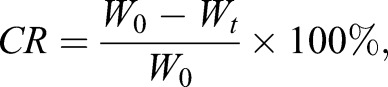
where *W*_g_ was the dry mass (mg), and *L* was body length (mm). *W*_0_ and *W*_t_ were the absolute contents of energy substances (mg ind^−1^) in the control group (day 0) and treatment group (60 days of starvation), respectively.

### Measurement of swimming performance and fin area

The experimental apparatus to measure the swimming performance was designed according to the device of McIntire et al. (1964); we further improvised the cuboid glass tank (100 cm long, 30 cm wide, and 20 cm high) with a swimming tunnel (40 cm long and 15 cm wide). The depth of water was 16 cm during the measurement, and the velocity in the tank was measured by the electromagnetic flow sensor (Model Starflow 6526, Australia).

In each replicate, a group of 20 fishes from the second group were used to measure the *U*_burst_, and the other 20 for *U*_crit_. According to a previous study, more than 20 samples were used to obtain an accurate average of the ability of the animal movement (Adolph and Pickering, 2008); thus, the sample size (*N*=60, three replicates and each replicate contained 20 fishes) in our study was sufficient to obtain the average rate of swimming performance. After measuring the *U*_burst_ and *U*_crit_, 20 fishes selected randomly from the group of 40 fishes were mildly anesthetized (50 mg l^−1^-buffered MS-222) in each replicate and assessed for fin areas. The body and fins of these fishes were fully expanded, and then the body length was measured nearest to 0.01 mm; subsequently, the expanded fins were photographed (Japan, Nikon D90). The images of the fins were analyzed by Image-Pro Plus (Media Cybernetics 6.0, USA) to evaluate the areas of pectoral and caudal fins.

### Measurement of *U*_burst_

The *U*_burst_ was measured according to the method described by Reidy et al. (2000). Individual fishes were placed in the swimming tunnel for 30 min before the swimming performance test, and the water velocity was increased to one body length per second (BL s^−1^) to acclimatize the fish to the conditions of the water tunnel. Subsequently, the water velocity in the swimming tunnel was steadily increased at a rate of 3.0 cm s^−1^ min^−1^ until the fish was exhausted. After impinging on the downstream retaining screen for >20 s, despite repeated attempts to stimulate continuous swimming, the ability of the fishes was assessed.

### Measurement of *U*_crit_

The fish were placed, individually, in the swimming tunnel, and the velocity was set at 2.1 cm s^−1^ (1 BL s^−1^) for 30 min to allow the fish to acclimatize to the conditions of the water tunnel. The incremental speed was set at 1 BL s^−1^ and the time interval was set at 10 min, which was similar to the study by Plaut (2001) until the fish was exhausted. The criterion of fatigue was the same as that for *U*_burst_. *U*_crit_ was calculated by the following formula:
(4)
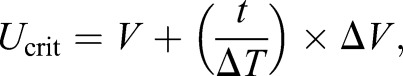
where *V* is the highest water velocity (cm s^−1^) sustained for an entire period of 10 min; Δ*V* is the velocity increment (2.1 cm s^−1^); *t* is the time elapsed at the fatigue velocity (min); Δ*T* is the prescribed constant period (10 min).

The results of *U*_burst_ and *U*_crit_ in the same fish may vary due to the differences in the rates of acceleration speed, Δ*V* and Δ*T*. Our results of *U*_burst_ and *U*_crit_ may not reflect the absolute speeds of *G*. *affinis* due to the lack of unified standard methods to measure the *U*_burst_ and Δ*V* and Δ*T* to measure *U*_crit_. However, we did not aspire to measure the absolute speeds, rather the relative differences in *U*_burst_ and *U*_crit_ between genders and starvation time. Thus, our results on swimming ability provided a consistent comparison because of the acceleration speed rates, Δ*V* and Δ*T*, in the swimming performance test at the same level (Belk and Tuckfield, 2010).

### Statistical analysis

Data are presented as means±s.e.m. Significant differences among different treatments were tested by two-way analysis of variance (two-way ANOVA) and further compared by one-way analysis of variance (one-way ANOVA) and Duncan's multiple comparison tests. The analysis of swimming performances was tested by analysis of covariance (ANCOVA), wherein the body lengths were used as a covariate. All statistical tests were conducted using SPSS 17.0 (SPSS Inc., USA).

## References

[BIO022822C1] Adolph S. C. and Pickering T. (2008). Estimating maximum performance: effects of intraindividual variation. *J. Exp. Biol.* 211, 1336-1343. 10.1242/jeb.011296 18375858

[BIO022822C2] Belk M. C. and Tuckfield R. C. (2010). Changing costs of reproduction: age-based differences in reproductive allocation and escape performance in a livebearing fish. *Oikos* 119, 163-169. 10.1111/j.1600-0706.2009.17742.x

[BIO022822C3] Bone Q., Kiceniuk J. and Jones D. R. (1978). Role of different fiber types in fish myotomes at intermediate swimming speeds. *U.S. Fish Wildl. Serv. Fish. Bull.* 76, 691-699.

[BIO022822C4] Brett J. R. (1972). The metabolic demand for oxygen in fish, particularly salmonids, and a comparison with other vertebrates. *Respir. Physiol.* 14, 151-170. 10.1016/0034-5687(72)90025-4 5042150

[BIO022822C5] Caiola N. and Sostoa A. D. (2005). Possible reasons for the decline of two native toothcarps in the Iberian Peninsula: evidence of competition with the introduced Eastern mosquitofish. *J. Appl. Ichthyol.* 21, 358-363. 10.1111/j.1439-0426.2005.00684.x

[BIO022822C6] Chen G. (2010). Interspecific relationship between the invasive species Gambusia affinis and the native endangered species Tanichthys albonubes. *PhD thesis*. Jinan University, Guangzhou.

[BIO022822C7] Chu C. Y. C. and Lee R. D. (2012). Sexual dimorphism and sexual selection: a unified economic analysis. *Theor. Popul. Biol.* 82, 355-363. 10.1016/j.tpb.2012.06.002 22699007 PMC3462896

[BIO022822C8] Deng S., Chen P. and Qiao D. (2007). *Introduction to Sport Physiology*. Beijing: Beijing Sport University Press.

[BIO022822C9] Elgar M. A. (1990). Evolutionary compromise between a few large and many small eggs: comparative evidence in teleost fish. *Oikos* 59, 283-287. 10.2307/3545546

[BIO022822C10] Faria A. M., Muha T., Morote E. and Chícharo M. A. (2010). Influence of starvation on the critical swimming behaviour of the Senegalese sole (*Solea senegalensis*) and its relationship with RNA/DNA ratios during ontogeny. *Sci. Mar.* 75, 87-94. 10.3989/scimar.2011.75n1087

[BIO022822C11] Jobling M. (1980). Effects of starvation on proximate chemical composition and energy utilization of plaice, *Pleuronectes platessa* L. *J. Fish Biol.* 17, 325-334. 10.1111/j.1095-8649.1980.tb02766.x

[BIO022822C12] Kieffer J. D. (2000). Limits to exhaustive exercise in fish. *Comp. Biochem. Physiol. A Mol. Integr. Physiol.* 126, 161-179. 10.1016/S1095-6433(00)00202-6 10938136

[BIO022822C13] Kieffer J. D. and Tufts B. L. (1998). Effects of food deprivation on white muscle energy reserves in rainbow trout (*Oncorhynchus mykiss*): the relationships with body size and temperature. *Fish Physiol. Biochem.* 19, 239-245. 10.1023/A:1007759407275

[BIO022822C14] Killen S. S., Marras S. and McKenzie D. J. (2014). Fast growers sprint slower: effects of food deprivation and re-feeding on sprint swimming performance in individual juvenile European sea bass. *J. Exp. Biol.* 217, 859-865. 10.1242/jeb.097899 24265431

[BIO022822C15] Kutty M. N. (1978). Ammonia quotient in sockeye salmon (*Oncorhynchus nerka*). *J. Fish. Res. Bd. Canada* 35, 1003-1005. 10.1139/f78-162

[BIO022822C16] Langerhans R. B. (2009). Trade-off between steady and unsteady swimming underlies predator-driven divergence in *Gambusia affinis*. *J. Evol. Biol.* 22, 1057-1075. 10.1111/j.1420-9101.2009.01716.x 21462405

[BIO022822C17] Langerhans R. B., Layman C. A., Shokrollahi A. M. and DeWitt T. J. (2004). Predator-driven phenotypic diversification in *Gambusia affinis*. *Evolution* 58, 2305-2318. 10.1111/j.0014-3820.2004.tb01605.x 15562692

[BIO022822C18] Langerhans R. B., Layman C. A. and DeWitt T. J. (2005). Male genital size reflects a tradeoff between attracting mates and avoiding predators in two live-bearing fish species. *Proc. Natl. Acad. Sci. USA* 102, 7618-7623. 10.1073/pnas.0500935102 15894618 PMC1140428

[BIO022822C19] Li D., Wei X. L., Lin X. T., Xu Z. N. and Mu X. P. (2015). Effects of exercise training on carbohydrate and lipid catabolism in the swimming muscles of Nile tilapia (Oreochromis niloticus). *J. Anim. Physiol. Anim. Nutr. (Berl.)* 99, 893-898. 10.1111/jpn.12300 25736102

[BIO022822C20] Li J., Lin X., Zhou C., Zeng P., Xu Z. and Sun J. (2016). Sexual dimorphism and its relationship with swimming performance in *Tanichthys albonubes* under laboratory conditions. *Chin. J. Appl. Eco. (China)* 27, 1639-1646. 10.13287/j.1001-9332.201605.028 29732827

[BIO022822C21] Lin H. (2011). *Fish Physiology*. Guangzhou: Zhongshan University Press.

[BIO022822C22] Liu M., Lin X., Xu Z., Xu C. and Yao D. (2014). Influences of fins amputation on swimming ability of *Tanichthy albonubes*. *Chin. J. Zool. (China)* 49, 930-937.

[BIO022822C23] Machado C. R., Garofaloj M. A. R., Roselino J. E. S., Kettelhut I. C. and Migliorini R. H. (1988). Effects of starvation, refeeding, and insulin on energy-linked metabolic processes in catfish (*Rhamdia hilarii*) adapted to a carbohydrate-rich diet. *Gen. Comp. Endocrinol.* 71, 429-437. 10.1016/0016-6480(88)90272-9 3056774

[BIO022822C24] Marras S., Claireaux G., McKenzie D. J. and Nelson J. A. (2010). Individual variation and repeatability in aerobic and anaerobic swimming performance of European sea bass, *Dicentrarchus labrax*. *J. Exp. Biol.* 213, 26-32. 10.1242/jeb.032136 20008358

[BIO022822C25] Martinez M., Bédard M., Dutil J. D. and Guderley H. (2004). Does condition of Atlantic cod (*Gadus morhua*) have a greater impact upon swimming performance at Ucrit or sprint speeds? *J. Exp. Biol.* 207, 2979-2990. 10.1242/jeb.01142 15277553

[BIO022822C26] McIntire C. D., Garrison R. L., Phinney H. K. and WARREN C. E. (1964). Primary production in laboratory streams. *Limnol. Oceanogr.* 9, 92-102. 10.4319/lo.1964.9.1.0092

[BIO022822C27] Osachoff H. L., Osachoff K. N., Wickramaratne A. E., Gunawardane E. K., Venturini F. P. and Kennedy C. J. (2014). Altered burst swimming in rainbow trout Oncorhynchus mykiss exposed to natural and synthetic oestrogens. *J. Fish Biol.* 85, 210-227. 10.1111/jfb.12403 24930959

[BIO022822C28] Oufiero C. E. and Garland T. (2009). Repeatability and correlation of swimming performances and size over varying time-scales in the guppy (*Poecilia reticulata*). *Funct. Ecol.* 23, 969-978. 10.1111/j.1365-2435.2009.01571.x

[BIO022822C29] Plaut I. (2000). Effects of fin size on swimming performance, swimming behaviour and routine activity of zebrafish *Danio rerio*. *J. Exp. Biol.* 203, 813-820. 10648223 10.1242/jeb.203.4.813

[BIO022822C30] Plaut I. (2001). Critical swimming speed: its ecological relevance. *Comp. Biochem. Physiol. A Mol. Integr. Physiol.* 131, 41-50. 10.1016/S1095-6433(01)00462-7 11733165

[BIO022822C31] Plaut I. (2002). Does pregnancy affect swimming performance of female Mosquitofish, *Gambusia affinis*? *Funct. Ecol.* 16, 290-295. 10.1046/j.1365-2435.2002.00638.x

[BIO022822C32] Pyke G. H. (2005). A review of the biology of *Gambusia affinis* and *G. holbrooki*. *Rev. Fish Biol. Fish.* 15, 339-365. 10.1007/s11160-006-6394-x

[BIO022822C33] Reidy S. P., Kerr S. R. and Nelson J. A. (2000). Aerobic and anaerobic swimming performance of individual Atlantic cod. *J. Exp. Biol.* 203, 347-357. 10607544 10.1242/jeb.203.2.347

[BIO022822C34] Richards J. G., Mercado A. J., Clayton C. A., Heigenhauser G. J. F. and Wood C. M. (2002). Substrate utilization during graded aerobic exercise in rainbow trout. *J. Exp. Biol.* 205, 2067-2077. 12089210 10.1242/jeb.205.14.2067

[BIO022822C35] Rios F. S., Kalinin A. L. and Rantin F. T. (2002). The effects of long-term food deprivation on respiration and haematology of the neotropical fish Hoplias malabaricus. *J. Fish Biol.* 61, 85-95. 10.1111/j.1095-8649.2002.tb01738.x

[BIO022822C36] Simpkins D. G., Hubert W. A., Del Rio C. M. and Rule D. C. (2003). Physiological Responses of Juvenile Rainbow Trout to Fasting and Swimming Activity: Effects on Body Composition and Condition Indices. *Trans. Am. Fish. Soc.* 132, 576-589. 10.1577/1548-8659(2003)132<0576:PROJRT%2.0.CO;2

[BIO022822C37] Sinclair E. L. E., Ward A. J. W. and Seebacher F. (2011). Aggression-induced fin damage modulates trade-offs in burst and endurance swimming performance of mosquitofish. *J. Zool.* 283, 243-248. 10.1111/j.1469-7998.2010.00776.x

[BIO022822C38] Stirling H. P. (1976). Effects of experimental feeding and starvation on the proximate composition of the European bass *Dicentrarchus labrax*. *Mar. Biol.* 34, 85-91. 10.1007/BF00390791

[BIO022822C39] Tu Z., Yuan X., Han J., Shi X., Liu G., Liu D. and Huang Y. (2011). Research advances on fish swimming capability. *Resour. Environ. Yangtze Basin (China)* 20, 59-65.

[BIO022822C40] Urzúa A., Guerao G., Cuesta J. A., Rotllant G., Estévez A. and Anger K. (2013). The bioenergetic fuel for non-feeding larval development in an endemic palaemonid shrimp from the Iberian Peninsula, Palaemonetes zariquieyi. *Mar. Freshw. Behav. Physiol.* 46, 381-397. 10.1080/10236244.2013.857067

[BIO022822C41] Van Damme R., Entin P., Vanhooydonck B. and Herrel A. (2008). Causes of sexual dimorphism in performance traits: a comparative approach. *Evol. Ecol. Res.* 10, 229-250.

[BIO022822C42] Ward D. L., Schultz A. A. and Matson P. G. (2003). Differences in swimming ability and behavior in response to high water velocities among native and nonnative fishes. *Environ. Biol. Fishes* 68, 87-92. 10.1023/A:1026031128486

[BIO022822C43] Weber J.-M. (2011). Metabolic fuels: regulating fluxes to select mix. *J. Exp. Biol.* 214, 286-294. 10.1242/jeb.047050 21177948

[BIO022822C44] Wilmore J. H. and Costill D. L. (1994). *Physiology of Sport and Exercise*. Champaign, IL: Human Kinetics.

[BIO022822C45] Yan Y., Chen Y. and Tao J. (2009). Ecological invasion of *Gambusia affinis*: a review. *Chin. J. Ecol. (China)* 28, 950-958.

[BIO022822C46] Yan Y.-Z., Zhan Y.-J., Chu L., Chen Y.-F. and Wu C.-H. (2010). Effects of stream size and spatial position on stream-dwelling fish assemblages. *Acta Hydrobiol. Sin. (China)* 34, 1022-1030. 10.3724/SP.J.1035.2010.01022

[BIO022822C47] Yeh M. F., Ho C. H. and Lee M. A. (2010). Critical swimming speeds and maximum sustainable swimming speeds of the minnows *Acrossocheilus paradoxus* and *Varicorhinus barbatulus* in comparison to the burst swimming speeds. *J. Fish. Soc. Taiwan* 37, 49-63.

[BIO022822C48] Yuan X. and Luo G. (2003). A brief review for ecological studies on hyporcheic zone of stream ecosystem. *Acta Ecol. Sin. (China)* 23, 956-964.

[BIO022822C49] Zheng W. and Pan J. (1985). Study on the reproductive property of *Gambusia affinis*. *Zoolog. Res. (China)* 6, 227-231.

[BIO022822C50] Zhou C., Wang Z., Lin X., Xu Z., Li J., Yang Y. and Zeng P. (2016). Temporal and spatial variations of *Tanichthys albonubes* population in a stream and the main affecting factors. *Sichuan J. Zool. (China)* 35, 344-350.

[BIO022822C51] Zhu Z., Zeng X., Lin X., Xu Z. and Sun J. (2015). Effects of ration levels on growth and reproduction from larvae to first-time spawning in the female *Gambusia affinis*. *Int. J. Mol. Sci.* 16, 5604-5617. 10.3390/ijms16035604 25768343 PMC4394495

[BIO022822C52] Zhu Z., Song B., Lin X. and Xu Z. (2016). Effect of sustained training on glycolysis and fatty acids oxidation in swimming muscles and liver in juvenile tinfoil barb Barbonymus schwanenfeldii (Bleeker, 1854). *Fish Physiol. Biochem.* 42, 1807-1817. 10.1007/s10695-016-0259-6 27387319

